# Incidence and risk factors of hypothyroidism after treatment for early breast cancer: a population-based cohort study

**DOI:** 10.1007/s10549-023-07184-8

**Published:** 2023-12-07

**Authors:** Evangelos Digkas, Daniel Robert Smith, Anna-Karin Wennstig, Alexios Matikas, Eva Tegnelius, Antonios Valachis

**Affiliations:** 1 Department of Immunology, Genetics and Pathology, Uppsala University, Uppsala, Sweden; 2 Department of Oncology, Uppsala University Hospital, Uppsala, Sweden; 3 Clinical Epidemiology and Biostatistics, School of Medical Sciences, Örebro University, Örebro, Sweden; 4 Department of Surgical and Perioperative Sciences, Umeå University, Umeå, Sweden; 5 Department of Oncology, Sundsvall Hospital, Sundsvall, Sweden; 6 Department of Oncology/Pathology, Karolinska Institute, Stockholm, Sweden; 7 Breast Center, Karolinska Comprehensive Cancer Center and Karolinska University Hospital, Stockholm, Sweden; 8 Department of Oncology, Faculty of Medicine and Health, Örebro University, Örebro, Sweden

**Keywords:** Hypothyroidism, Breast cancer, Population-based, Radiation therapy, Chemotherapy, Endocrine therapy

## Abstract

**Purpose:**

An increased incidence of hypothyroidism among breast cancer survivors has been observed in earlier studies. The impact of the postoperative treatment modalities and their potential interplay on hypothyroidism development needs to be studied.

**Methods:**

We conducted a population- and registry-based study using the Breast Cancer Data Base Sweden (BCBaSe) including females diagnosed with breast cancer between 2006 and 2012. In total, 21,268 female patients diagnosed with early breast cancer between 2006 and 2012, with no previous prescription of thyroid hormones and no malignant diagnosis during the last ten years before breast cancer diagnosis, were included in the final analysis.

**Results:**

During the follow-up (median follow-up time 7.9 years), 1212 patients (5.7%) developed hypothyroidism at a median time of 3.45 years from the index date. No association of the systemic oncological treatment in terms of either chemotherapy or endocrine therapy and hypothyroidism development could be identified. A higher risk (HR 1.68;95% CI 1.42–1.99) of hypothyroidism identified among patients treated with radiation treatment of the regional lymph nodes whereas no increased risk in patients treated only with radiation therapy to the breast/chest wall was found (HR 1.01; 95% CI 0.86–1.19). The risk of hypothyroidism in the cohort treated with radiotherapy of the regional lymph nodes was present irrespective of the use of adjuvant chemotherapy treatment.

**Conclusions:**

Based on the results of our study, the implementation of hypothyroidism surveillance among the breast cancer survivors treated with radiotherapy of the regional lymph nodes can be considered as reasonable in the follow-up program.

## Introduction

Breast cancer has become the leading malignant diagnosis since 2020 with more than two million new cases among women globally [1], while it is estimated that in 2040 about 3 million new cases will be diagnosed [2]. The rising incidence trend along with the reduced mortality over time among breast cancer patients result in an increased number of breast cancer survivors that is expected to rise in future. As a result, the understanding and recognition of long-term sequelae related to breast cancer diagnosis per se or treatment is essential to be able to adopt adequate strategies for prevention, early detection, and management of these conditions.

Hypothyroidism is relatively common in the general population with a prevalence of up to 5.3% with most of the cases being subclinical and consequently diagnosed as an accidental finding [3]. Although patients in the general population diagnosed with hypothyroidism seem to face an increased risk for multiple morbidities and higher mortality [4], hypothyroidism either at breast cancer diagnosis or diagnosed after breast cancer does not seem to affect cancer-specific or all-cause mortality [5].

In breast cancer patients, radiation therapy to the supraclavicular lymph nodes has been associated with increased risk for hypothyroidism [6, 7]. Some studies suggest a potential impact of systemic treatment to the risk of hypothyroidism in breast cancer patients as well [8–14]. However, the results from these studies are contradictory and inconclusive due to the small sample size [8, 10, 14], the lack of population-based data, and the lack of information on specific treatment approaches [10, 11, 13].

It is, therefore, essential to investigate the incidence and risk factors of hypothyroidism after breast cancer diagnosis in a population-based cohort including all relevant information on different treatment strategies, both systemic and locoregional.

In this population-based retrospective cohort study, we aimed to investigate the impact of different adjuvant treatment strategies on the risk for developing hypothyroidism after breast cancer diagnosis.

## Methods

### Study design, data source, and patient cohort

In this population- and register-based cohort study, we used the research database Breast Cancer Data Base Sweden (BCBaSe) as data source. BcBaSe is a database derived from the linkage of the National Quality Registry for Breast Cancer covering three healthcare regions in Sweden (Stockholm-Gotland, Uppsala-Örebro, North Region), which is corresponding to nearly 60% of Swedish population, with other national Registries of interest (the Prescribed Drug Registry, the National Patient Registry, and the Swedish cause of death registry).

Through BCBaSe, we identified all patients with non-metastatic breast cancer diagnosed at least one year after initiation of Prescribed Drug Registry (launched on July 1, 2005). Patients with bilateral breast cancer were also included. The date of breast cancer diagnosis was served as the index date.

We excluded men with breast cancer, patients with prior exposure to thyroid hormones (ATC-code: H03AA) before index date, and patients with prior cancer diagnosis (ICD-codes: C00-C14, C30-C32, C34, C50, C73) up to 10 years before index date.

### Outcomes and definitions

The primary outcome was the frequency of new onset hypothyroidism in patients diagnosed with non-metastatic breast cancer. New onset hypothyroidism was defined as initiation of thyroid hormones (ATC-code: H03AA) from index date plus 90 days and onwards with at least two prescriptions irrespective of defined daily dose (DDD) without any prescription of antithyroid preparations (ATC-code: H03B) or surgical procedure to thyroid gland (ICD-10 codes: BAA40, BAA50, BAA60, and BAA99) at any time during the follow-up.

Secondary outcome was the identification of potential risk factors for development of hypothyroidism after breast cancer diagnosis with special interest in different oncological treatment strategies.

### Data collection

The following data were extracted from the BCBaSe: age at diagnosis, date at breast cancer diagnosis, region, menopausal status, relevant autoimmune comorbidities that could be associated with increased risk for hypothyroidism and had a prevalence in the cohort enabling statistical analyses (diabetes mellitus type 1, seropositive or seronegative rheumatoid arthritis, systemic lupus erythematosus), any exposure to medications that can lead to hypothyroidism (lithium with ATC-code N05AN01; amiodarone with ATC-code C01BD01) after index date, T status, N status, histology, ER (estrogen receptor)-status, PgR (progesterone-receptor)- status, tumor grade, Her2-status (with IHC or FISH); type of primary surgery, oncological treatment including chemotherapy, radiotherapy, endocrine therapy, and anti-HER2 treatment.

In terms of oncological treatment, the retrieved information from BCBaSe corresponds to planned treatment. Due to a considerably high percentage of missing data regarding HER2 status and anti-HER2 treatment, we did not include this treatment strategy in our analyses. Regarding radiotherapy, the available information through BCBaSe was whether patients were planned for radiotherapy or not and the planned irradiated target as breast/chest wall, regional lymph nodes, or both. According to the Swedish guidelines during the study period, radiation therapy to regional lymph nodes included axillary stations level II to IV but not internal mammary nodal stations.

### Statistical analysis

Summary statistics are presented as frequencies and percentages for categorical variables and medians and interquartile ranges for continuous variables. Time to diagnosis of hypothyroidism was analyzed using Cox proportional hazards models stratified on region, with breast cancer diagnosis date representing the index date. We allowed for competing risks by censoring in the event of emigration or death. Maximum follow-up date was 31 December 2018. Treatment variables were categorical and included: radiation therapy, chemotherapy and endocrine therapy. Separate univariate Cox models were fitted to each of these treatment variables to estimate unadjusted effects. A multivariable Cox model was used to estimate adjusted effects, which in addition to the above treatment variables included: exposure to amiodarone and lithium (any DDD) post index date; diagnoses of type 1 diabetes, rheumatoid arthritis with rheumatic factor, other rheumatoid arthritis, and systemic lupus erythematosus pre- or post- index date; and diagnosis date and age at diagnosis (continuous variables modeled as restricted cubic splines using three pre-specified knots). Effects were presented as Hazard Ratios (HR) and 95% Confidence Intervals (CI). The proportional hazards assumption was assessed using unique term and global Chi-squared hypothesis tests (*α* = 0.05) as well as plots of the Schoenfeld residuals. No violation of proportional hazards was detected, with the exception of amiodarone. We, therefore, fitted an additional multivariable model with the same parameterization as above, but also including an interaction between amiodarone and time to event, modeled as a restricted cubic spline with three pre-specified knots. We, then, computed cumulative incidence curves for radiotherapy (stratified on region) accounting for competing risks of emigration and death. All statistical analyses were performed in R studio version 2022.07.2, using R version 4.2.2 [15], relying heavily on the packages survival, tidycmprsk, and the tidyverse suite [16–18]

## Results

### Selection process and characteristics of patient cohort

**Fig. 1:**
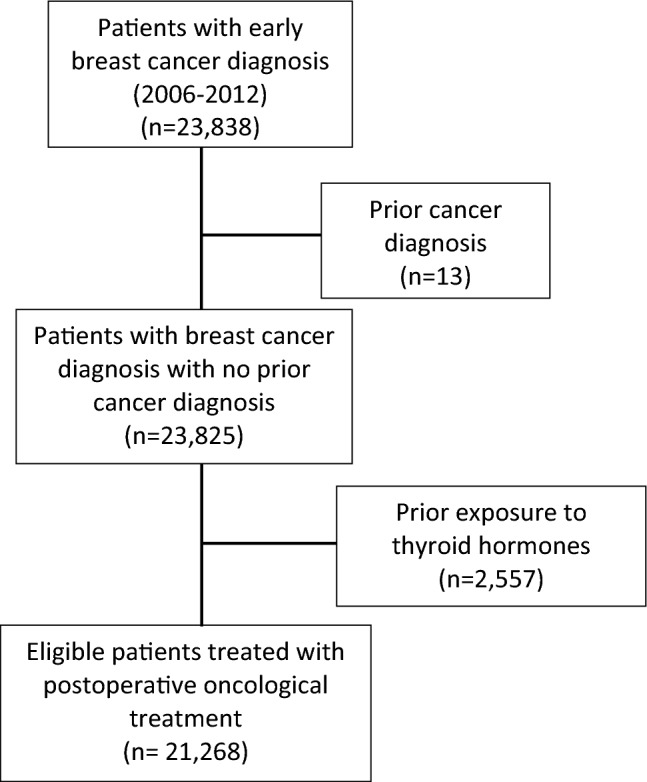
Flowchart diagram of study cohort selection

A flowchart of patient cohort selection process is illustrated in Fig. 1. Through the BCBaSe, 23,838 female patients with non-metastatic breast cancer were identified. After applying the exclusion criteria, 21,268 patients with breast cancer diagnosed between July 1st, 2006, and December 31st, 2012, were eligible for the study cohort (Fig. 1).

Patient-, tumor-, and treatment-related characteristics of patient cohort are presented in Table 1. The median age at diagnosis was 63 years (IQR 52–72 years). The percentage of patients treated with any adjuvant radiotherapy, endocrine therapy, and chemotherapy were 67.7%, 70.3%, and 31.8%, respectively. Regarding radiotherapy, the irradiated target included breast/chest wall only in 8953 patients (42.1%) whereas 5436 patients (25.6%) received radiotherapy to the regional lymph nodes as well.

**Table 1:** Baseline characteristics of the included population in the final analysis

	Number of patients n(%)
Total	21,268
Median follow-up time in years	7.9
Age at diagnosis	
< 40	916 (4.3%)
40–49	3192 (15.0%)
50–59	4600 (21.6%)
≥ 60	12,560 (59.1%)
Menopausal status	
Premenopausal	4378 (20.6%)
Postmenopausal	15,037 (70.7%)
Unknown	1853 (8.7%)
Region of residence	
Northern	3558 (16.7%)
Stockholm/Gotland	8786 (41.3%)
Uppsala/Örebro	8924 (42.0%)
Autoimmune disease as a comorbidity	
Diabetes mellitus type 1	549 (2.6%)
Systemic Lupus erythematosus	55 (0.3%)
Rheumatoid athritis	661 (3.1%)
None	20,003 (94.0%)
Treatment with risk for secondary hypothyroidism	
Lithium	87 (0.4%)
Amiodarone	58 (0.3%)
None	21,123 (99,3%)
Hypothyroidism during follow-up	
Yes	1212 (5.7%)
No	20,056 (94.3%)
Breast cancer characteristics	
Histology	
Ductal	16,355 (76.9%)
Lobular	1502 (7.1%)
Other	1022 (4.8%)
Unknown	2389 (11.2%)
Tumor size	
T1	12,368 (58.2%)
T2	6085 (28.6%)
T3	838 (3.9%)
Unknown	1977 (9.3%)
Lymph nodes metastatasis	
N0	12,408 (58.3%)
N1	4627 (21.8%)
N2–3	2069 (9.7%)
Unknown	2164 (10.2%)
Histological grade	
I	3250 (15.3%)
II	8020 (37.7%)
III	4783 (22.5%)
Unknown	5215 (24.5%)
ER status	
Positive	16,810 (79.0%)
Negative	2920 (13.7%)
Unknown	1538 (7.3%)
PR status	
Positive	13,892 (65.3%)
Negative	5726 (26.9%)
Unknown	1650 (7.8%)
HER2 status	
Positive	2328 (10.9%)
Negative	15,051 (70.8%)
Unknown	3889 (18.3%)
Breast cancer treatment	
Type of surgery	
Partial mastectomy	11,414 (53.7%)
Mastectomy	8145 (38.3%)
Unknown	1709 (8.0%)
Axillary surgery	
Axillary dissection	6296 (29.6%)
Sentinel node dissection	9411 (44.2%)
Unknown	5561 (26.2%)
Adjuvant chemotherapy	
Taxanes only	1088 (5.1%)
Anthracyclines only	2544 (11.9%)
Both taxanes and anthracyclines	3137 (14.8%)
None	14,499 (68.2%)
Endocrine therapy	
Aromatase inhibitors only	1634 (7.7%)
Tamoxifen only	8932 (42.0%)
Both tamoxifen och aromatase inhibitors	4383 (20.6%)
None	6319 (29.7%)
Adjuvant radiotherapy	
Breast/chest wall	8953 (42.1%)
Breast/chest wall and axillary & subclavian lymph nodes	5436 (25.6%)
None	6879 (32.3%)

### Development of hypothyroidism after breast cancer diagnosis

During the follow-up (median follow-up 7.9 years; IQR 6.1–10.1), 1212 patients (5.7%) developed hypothyroidism with a median time of 3.45 years (IQR 1.67–5.65 years) from index date to diagnosis of hypothyroidism.

### Impact of oncological treatment strategies on the risk for hypothyroidism

Table 2 presents the results from crude and adjusted analyses investigating the impact of different treatment strategies on the risk for hypothyroidism.

**Table 2:** Risk of hypothyroidism development among women diagnosed with early breast cancer between 2006 and 2012 in Sweden associated to the treatment modality calculated by the crude analysis and adjusted analysis

	Crude analysis			Adjusted analysis		
Treatment	HR	(95% CI)	*p*-value	HR	(95% CI)	*p*-value
Radiation therapy						
None						
Breast/chest wall	1.01	(0.87–1.17)	> 0.90	1.01	(0.86–1.19)	0.9
Breast/chest wall and regional lymph nodes	1.79	(1.54–2.08)	< 0.001	1.68	(1.42–1.99)	< 0.001
Chemotherapy						
None						
Taxanes only	1.60	(1.29–2.00)	< 0.001	1.20	(0.94–1.52)	0.15
Anthracyclines only	1.32	(1.11–1.56)	0.001	1.15	(0.96–1.38)	0.12
Both anthracyclines and taxanes	1.41	(1.21–1.66)	< 0.001	1.08	(0.90–1.30)	0.4
Endocrine therapy						
None						
Aromatase inhibitors only	1.12	(0.89–1.43)	0.3	0.98	(0.77–1.25)	0.9
Tamoxifen only	0.98	(0.85–1.14)	0.8	0.99	(0.85–1.15)	> 0.9
Both aromatase inhibitors and tamoxifen	1.24	(1.05–1.46)	0.011	1.09	(0.91–1.30)	0.3

No association between use of endocrine therapy and development of hypothyroidism was observed, irrespective of the type of endocrine therapy (aromatase inhibitors, tamoxifen, or sequential therapy). In terms of chemotherapy use, a potential association between chemotherapy use (irrespective of the type of chemotherapeutic regimen) and hypothyroidism could be suspected in crude analyses but it was not confirmed in adjusted analyses.

Regarding radiotherapy, patients treated with radiotherapy including regional lymph nodes were at higher risk for developing hypothyroidism (HR 1.68; 95% CI 1.42–1.99) whereas no such association was observed in patients treated with radiotherapy only to the breast / chest wall (HR 1.01; 95% CI 0.86–1.19). In this adjusted analysis, patients treated with radiotherapy were stratified based on the use of adjuvant chemotherapy or not without resulting in any statistically significant interaction (*p*-value = 0.710).

**Fig. 2:**
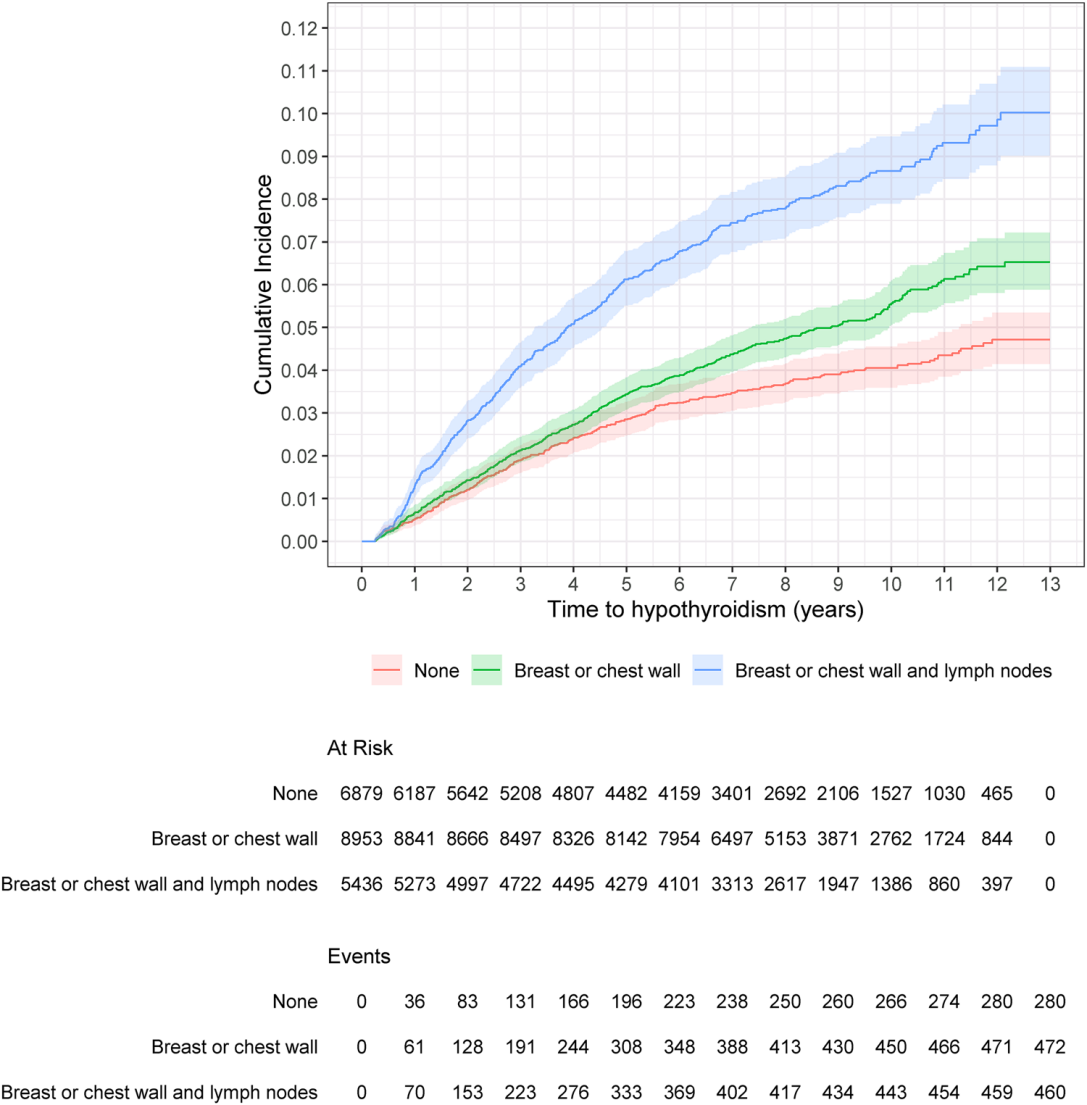
Cumulative incidence of hypothyroidism for different radiotherapy exposures, accounting for death and emigration as competing risks. Shaded regions are 95% confidence bands

By analyzing the cumulative incidence of hypothyroidism stratified by radiotherapy use, cumulative incidence at 5 years for patients without radiotherapy, those treated with radiotherapy only to breast / chest wall, and those treated with radiotherapy to regional lymph nodes was 2.8% (95% CI 2.5–3.3%), 3.4% (95% CI 3.1–3.8%), and 6.1% (95% CI 5.5–6.8%), respectively, whereas the corresponding cumulative incidence at 10 years was 4.1% (95% CI 3.6–4.6%), 5.6% (95% CI 5.1–6.1%), and 8.7% (95% CI 7.9–9.5%), respectively (Fig. 2).

## Discussion

In this population-based study, among breast cancer patients treated with multimodal approaches in the adjuvant setting, a higher risk for hypothyroidism was found only to those patients treated with radiotherapy including regional lymph nodes irrespective of the use of chemotherapy or not whereas no impact of systemic oncological treatment on hypothyroidism was observed. The considerably higher cumulative incidence of hypothyroidism in this patient group (8.7% in 10 years) supports the consideration of thyroid gland as organ-at-risk during treatment planning and the potential need for thyroid function screening as a part of follow-up strategy.

The higher risk for hypothyroidism in breast cancer patients treated with radiotherapy including regional lymph nodes is in accordance with previous studies [6, 7, 19]. The pathophysiological mechanism of this association, through direct thyroid cell injury or indirect due to damage in small thyroid vessels, has been described [20] and is further supported by dosimetric data showing higher mean radiation dose to the thyroid gland in patients treated with radiotherapy including regional lymph nodes compared to patients with radiotherapy only to breast / chest wall [19] and a dose-dependent association between dose to the thyroid gland and decreased thyroid volume [11]. The present study strengthens the current evidence by overcoming some of the main limitations of previous studies [21]. First, previous studies included a relatively small sample size [7, 22, 23], thus reducing their validity to provide reliable results on potential associations. Second, several studies lacked information on important risk factors for hypothyroidism, thus increasing the risk for confounding bias [7, 24]. Although we were not able to adjust for lifestyle factors as potential confounders, we could consider confounders related to comorbidities and other medications. Third, previous studies were prone to an upward biased estimation of incidence of hypothyroidism since they often did not account for competing events [25]. By applying suitable statistical approaches for competing events as emigration and death, our estimates might better reflect the expected incidence of hypothyroidism in this clinical situation.

An additional strength of the present study is the inclusion of systemic oncological treatment approaches in the analyses enabling the interrogation of the interplay and potential synergistic effect of systemic therapy and radiation therapy on thyroid dysfunction. Neither endocrine therapy nor chemotherapy were found to be associated with increased risk for hypothyroidism whereas radiotherapy including regional lymph nodes remained a risk factor when accounting for systemic treatment approaches.

In terms of endocrine therapy, a recent systematic review suggested a mild and transient thyroid dysfunction in patients treated with tamoxifen [8]. However, this transient thyroid dysfunction seems not to be translated into a clinical hypothyroidism that would need thyroid hormone replacement therapy according to our study results. In fact, our definition of hypothyroidism was solely based on the prescription of thyroid hormones after breast cancer diagnosis, thus reflecting only the occurrence of hypothyroidism that would be captured and treated.

Considering the potential impact of chemotherapy on the development of hypothyroidism, some preclinical evidence supports a potential association, mainly through altering the levels of thyroid hormone-binding proteins [26] or serving as radiosensitizer in the thyroid gland [27] does exist. However, the clinical evidence on this potential association is rather limited and uncertain with few studies suggesting some transient changes in thyroid hormone levels with questionable clinical relevance [28]. Our study results did not support either the potential direct impact of chemotherapy on hypothyroidism (no increased risk for hypothyroidism in chemotherapy-treated patients) or the hypothesis that chemotherapy might serve as radiosensitizer in the thyroid gland (similar magnitude of risk between patients treated with radiotherapy in regional lymph nodes with and without chemotherapy).

In our study cohort, the cumulative incidence of hypothyroidism at 10 years was found to be 8.7% in the cohort treated with radiotherapy including regional lymph nodes. It should be noted that this estimate is difficult to be compared with previous studies because of the various definitions used across the studies and the fact that we applied suitable statistical approaches to deal with competing events for calculation of cumulative incidence. Our estimate reflects the incidence of the occurrence of a clinical hypothyroidism required thyroid hormone replacement therapy but it does not consider overt hypothyroidism or hypothyroidism not requiring therapy. As a result, misclassification bias cannot be mitigated through our study design or method and should be considered as a potential limitation. However, one could argue that capturing hypothyroidism requiring therapy is a clinically relevant outcome with stronger clinical impact compared to subclinical hypothyroidism.

Apart from the above-mentioned limitations of misclassification bias and lack of information about lifestyle-related confounders, some additional limitations should be discussed. First, we lack volumetric and dosimetric data regarding radiotherapy. As a result, our results rely on the intended irradiated volume (breast/chest wall ± regional lymph nodes) and the translation of the national guidelines on target volume delineation during the study period. According to the guidelines, target volume for regional lymph nodes included level IV (supraclavicular area) using the caudal to the cricoid cartilage as cranial border. As radiation techniques are evolving and target volume delineation strategies are refined, 2015 ESTRO-guidelines recommended the subclavian artery arch for the cranial edge of level IV [29], thus reducing the mean dose to thyroid gland compared to the previous recommendation [19]. Our study results can, therefore, be applied to patients treated with radiotherapy to regional lymph nodes including level IV up to the cricoid cartilage whereas the potential impact of the 2015 ESTRO-recommendation on the risk of hypothyroidism should be further studied. An additional limitation is the fact that the treatment-related information captured in the BCBaSe refers to the planned treatment. Although some discrepancy between planned and given treatment can be anticipated, this proportion is expected to be low. Furthermore, we lack information on adherence to endocrine therapy among patients treated with this treatment strategy. However, two recent Swedish studies investigating the adherence to adjuvant endocrine therapy showed that more than 80% of the patients followed the prescribed medication 5 years after initiation [30, 31], thus supporting the notion that most of the patients included in our study cohort might also have followed the prescribed treatment. Finally, our study cohort lacks reliable information on trastuzumab use so this treatment strategy was not included into the analyses. Considering some case reports suggesting a potential association between trastuzumab and thyroid dysfunction [32], further studies including patients treated with anti-HER2 treatment strategies are required.

In conclusion, our study results confirm the increased risk of hypothyroidism in breast cancer patients treated with radiotherapy including regional lymph nodes as target volume. We found no association between systemic oncological treatment as endocrine therapy or chemotherapy and risk of hypothyroidism. Given the considerably high cumulative incidence of clinical hypothyroidism requiring thyroid hormone replacement therapy in the cohort of irradiated patients, it is motivated to incorporate thyroid gland as organ-at-risk during the treatment planning in order to gain detailed information on the radiation dose to the thyroid and evaluate dose–response associations. The high cumulative incidence also suggests a potential role of implementing regular monitoring of thyroid function as a part of the follow-up strategy in patients treated with radiotherapy including regional lymph nodes.

## Data Availability

The Breast Cancer DataBase Sweden (BCBaSe) cohort was used in this study. It is a population-based database that comprises all new cases of invasive breast cancer in women from 1992 to 2012 in three Swedish health care regions. The cohort was linked to a number of national population-based registries. Since BCBaSe contains sensitive health information, it cannot be published in open repositories. Those interested in data from BCBaSe should contact the corresponding author.
